# A Metacognitive Perspective on Mindfulness: An Empirical Investigation

**DOI:** 10.1186/s40359-015-0081-4

**Published:** 2015-07-14

**Authors:** Stian Solem, Susanne Semb Thunes, Odin Hjemdal, Roger Hagen, Adrian Wells

**Affiliations:** Department of psychology, Norwegian University of Science and Technology, 7491 Trondheim, Norway; St. Olavs University Hospital, Trondheim, Norway; Department of Clinical Psychology, University of Manchester, Manchester, England

**Keywords:** Metacognition, Mindfulness, Depression, Anxiety, OCD

## Abstract

**Background:**

The primary aim of this study was to explore how metacognition, as implicated in Wells and Matthews’ metacognitive theory of emotional disorder, might relate to the concept of mindfulness, and whether metacognition or mindfulness best predicted symptoms of emotional disorder.

**Methods:**

Data was collected from 224 community controls on the Five Facet Mindfulness Questionnaire (FFMQ), the Metacognitions Questionnaire-30 (MCQ-30), the Patient Health Questionnaire 9-item (PHQ-9), the Generalized Anxiety Disorder 7-item (GAD-7), and the Obsessive-Compulsive Inventory Revised (OCI-R).

**Results:**

The MCQ-30 and FFMQ subscales constituted two latent factors which appeared to assess metacognition and mindfulness. The FFMQ subscales *nonjudging of inner experience* and *acting with awareness* loaded on metacognition, while *observing, nonreacting to inner experience* and *describing* formed a unique mindfulness factor. Metacognition correlated strongly with symptoms of depression, anxiety and obsessive-compulsive disorder. Regression analyses found metacognition to be an important predictor of symptoms explaining between 42 % and 49 % of the variance when controlling for age and gender, while mindfulness was a weaker predictor explaining between 0 % and 2 % of the variance in symptoms.

**Conclusions:**

The structure amongst scales and the pattern of correlations with symptoms were generally consistent with the metacognitive theory which focuses on metacognitive beliefs, enhancing awareness of thoughts and disengaging extended processing.

## Background

Psychological treatments develop in parallel and some of their therapeutic interventions and constructs may overlap or share elements. Exploring and clarifying the relation between potentially related constructs may be useful because it may contribute to a better understanding of similarities and differences, which may be essential in both understanding and developing treatment interventions.

Two recent approaches to treatment are represented by the metacognitive model (Wells and Matthews 1994, Wells 2009) and mindfulness-based therapies (Kabat-Zinn 1994). Each use strategies aimed at directly changing experiential awareness, but do so in different ways. The metacognitive perspective is founded on a specific information processing model of attention and emotion whilst mindfulness draws on Buddhist principles and techniques. It has been argued that effective experiential techniques might be grounded in the metacognitive model in order to conceptualize what mindfulness is and how it should be developed from an information processing perspective (Wells 2005). More specifically, in the metacognitive model, psychological disorder is related to a style of reacting to thoughts with sustained processing in the form of worry, rumination, fixation of attention on threat and maladaptive coping behaviors, collectively referred to as the Cognitive Attentional Syndrome (CAS; Wells and Matthews 1994, 1996). The CAS is the result of the influence of metacognitive beliefs on cognitive processing. For example, the metacognitive belief that thoughts are dangerous and/or uncontrollable leads to sustained negative thinking and entanglement with them.

Theory and research on metacognition evolved from developmental- and cognitive psychology (e.g., Flavell 1979; Nelson 1996; Nelson and Narens 1994). Metacognition was originally defined as knowledge or beliefs about thinking and strategies used to regulate and control thinking processes (Flavell, 1979). As such metacognition refers to cognition applied to cognition. Metacognition has since been developed as a basis for understanding and treating psychological disorders (Wells and Matthews, 1994; 1996). In this metacognitive theory of emotional disorders it is argued that disorder is associated with a non-specific style of thinking termed CAS. According to the theory, much of the knowledge on which processing depends is metacognitive in nature. So the activation and persistence of the CAS in response to stressors is dependent on maladaptive metacognitive knowledge (beliefs). When we refer to metacognitions in the current study, we refer to metacognitions as specified in this metacognitive model of emotional disorders and as operationalized by the Metacognitions Questionnaire-30 (MCQ-30). For a detailed theoretical discussion on metacognition in general (not restricted to Wells’ metacognitive model) and mindfulness confer Jankowski and Holas (2014).

Within a different theoretical framework, mindfulness, has gained extensive import into the therapeutic domain through the adoption of meditation practices. In this tradition mindfulness has been defined as “the awareness that emerges through paying attention on purpose, in the present moment, and nonjudgmentally to the unfolding of experience moment by moment” (Kabat-Zinn 2003, p. 145). As noted by Jankowski and Holas (2014) the abundance of meanings related to the term mindfulness makes it difficult to define. In the current study mindfulness is understood and operationalized according to the FFMQ. According to FFMQ mindfulness is a multifaceted construct which could be defined as: attending to internal and external experiences, labelling internal experiences with words, attending to the present moment, taking a nonjudging stance toward thoughts and feelings, and not getting caught up in thoughts and feelings.

To practice mindfulness is to be aware of what is going on in the present moment. It is the opposite of acting on “autopilot”, where the present moment is biased by routinized and habitual thoughts and feelings. While practicing mindfulness, a person will experience the present moment as the direct experience of the body and sensory input. Meditation is used as a tool to develop the state, or the skill of mindfulness. Emotional disorders could possibly be understood as a lack of mindfulness skills in the mindfulness-based approach. However, there is a lack of theory to offer a good explanation as to the mechanisms of mindfulness, but there are indications that mindfulness could be useful in managing cognitive vulnerability and reduce the chance of relapse in depression (Segal et al. 2013). In contrast, in the metacognitive model emotional disorders are seen as the result of fixed ways of coping with negative thoughts through repetitive negative thinking. Disorder does not result from a state of mindlessness or ‘autopilot’ instead it results from excessive motivated efforts to deal with negative thoughts with the CAS. Metacognitive therapy has developed specific techniques such as *detached mindfulness* (Wells and Matthews 1994, Wells 2005), which is a reaction to thoughts that is the opposite of the CAS, involving standing back and not reacting or trying to deal with them but remaining flexible with low levels of ideation. This ability is aimed to strengthen metacognitive beliefs such as knowing what a thought is and that it is a benign inner event that is separate from the control of action. See Wells (2005) for a closer description of detached mindfulness and its similarities and differences from mindfulness.

Recent analyses have attempted to differentiate and assess the components of mindfulness and link them meaningfully with different psychological factors. The concept of mindfulness has been operationalized with the Five Facet Mindfulness Questionnaire (FFMQ; Baer et al. 2006), based on a factor analysis of independently developed questionnaires for measuring mindfulness. A systematic review of instruments to measure self-reported mindfulness found that the FFMQ has the best psychometric properties (Park et al. 2013). The higher the scores on the FFMQ the better mental health the individual is thought to have. The five facets of FFMQ are: *observing, describing, acting with awareness, nonjudging of inner experience,* and *nonreacting to inner experience. Observing* includes attending to or noticing experiences, either internal or external such as cognitions, emotions or various sensory perceptions (e.g. “I pay attention to how my emotions affect my thoughts and behavior”). *Describing* refers to being able to put internal experiences into words (e.g. “I’m good at finding words to describe my feelings”)*. Acting with awareness* is the tendency to attend to what you’re doing at the moment, as opposed to “going on automatic pilot” (e.g. “When I do things, my mind wanders off and I’m easily distracted”)*. Nonjudging of inner experience* refers to being able to not evaluate thoughts and feelings as good or bad (e.g. “I criticize myself for having irrational or inappropriate emotions” [reverse scoring])*. Nonreacting to inner experience* refers to the ability to let thoughts and emotions come and go without being caught up in or reacting to them (e.g. “I watch my feelings without getting lost in them”) (Baer et al. 2008).

Baer et al. (2006) showed that *nonjudging of inner experience* and *acting with awareness* were the subscales most strongly correlated with psychological symptoms, neuroticism, thought suppression and difficulty regulating emotion, while *observing* and *describing* mostly showed no or weak correlations with these measures. Another study showed that *observing* and *describing* had no correlation to anxiety and depression, while the other three subscales showed weak correlations (Bohlmeijer et al. 2010). It has also been found that *nonjudging of inner experience* predicts lower levels of depression, anxiety and stress, while *acting with awareness* predicts lower levels of depression (Cash and Whittingham, 2010).

There are similarities and differences in the concepts used in metacognitive theory and in meditation derived practice. The concept of mindfulness as assessed with the FFMQ (Baer et al. 2006) may be related to the constructs of metacognitive beliefs as specified in metacognitive theory (Wells and Matthews 1994). For instance, items of the FFMQ (e.g. “When I do things my mind wanders off and I’m easily distracted”) appear to tap attitudes or beliefs about cognition. In metacognitive theory of psychological disorder metacognitive beliefs are assessed with the metacognitions questionnaire (MCQ; Cartwright-Hatton and Wells 1997; Wells and Cartwright-Hatton 2004). The MCQ has five subscales: *positive beliefs about worry* (e.g. “Worrying helps me cope”), *uncontrollability and danger* (e.g. “When I start worrying I cannot stop”), *need to control thoughts* (e.g. “I should be in control of my thoughts all of the time”), *cognitive confidence* (e.g. “I do not trust my memory”), and *cognitive self—consciousness* (e.g. “I am constantly aware of my thinking”).

There is both convergence and divergence in the items of the FFMQ and MCQ. Whilst the FFMQ and MCQ contain constructs with no apparent overlap, it is clear for example, that the FFMQ construct of *observing* overlaps with MCQ *cognitive self-consciousness* as they both relate to an internal focus of attention. However, in the metacognitive model *cognitive self-consciousness* should be positively related to troublesome symptoms, while the mindfulness construct of *observing* should be negatively related to troublesome symptoms. However, there might be differences between experienced and inexperienced meditators as they have different cerebral activation in response to stressors (e.g. Froeliger et al. 2012) and meditation experience could also affect the relationship between *observing* and well-being (Baer et al. 2006; 2008).”

It would be useful to establish whether metacognition correlates with mindfulness and whether mindfulness and metacognition subscales combine to form latent variables that might predict psychological distress. The primary aim of the current study was therefore to explore how metacognition as implicated in metacognitive theory of emotional disorder might relate to the broad concept of mindfulness. We also wanted to examine the latent structure among factors related to mindfulness and metacognitions and how this might relate to anxiety, depressive and obsessive-compulsive symptoms. Because the constructs may be related in different ways (or not at all) our goal was exploratory rather than testing a particular model fit, as we did not wish to impose constraints on any meaningful structure that could emerge. In addition to exploring the latent structure among subscales we examined, post hoc, the relationship between the latent factors and measures of psychopathology as a means of provisionally validating emergent constructs.

## Methods

### Participants and procedure

The sample was 224 Norwegian-speaking participants between the age of 18 and 67, with a mean age of 31.8 years (SD = 13.0). The sample consisted of 75 (33.5 %) men and 149 (66.5 %) women. In this sample, 39.8 % were employed either part time or full time, 45.1 % were full time students, 1.8 % were part time students, and 13.4 % received disability benefits or disability insurance or were retired or unemployed. Also, 34.4 % was single, 21.4 % was in a relationship, 38.8 % was married or cohabiting, and 5.4 % was divorced or separated. The data was anonymously collected online through posts on social media and on public online discussion forums where people were encouraged to answer and to share a link to the questionnaires. The majority of participants recruited through social media were from the network of a psychology graduate student, while the discussion forums were related to psychological health. Anyone over the age of 18 could participate, and 224 people completed all five questionnaires. The study was submitted to the Regional Committee for Medical and Health Research Ethics in Norway for approval. The ethics committee decided that an approval was not necessary as the participants responses were anonyomous. The study was approved by the Norwegian Social Science Data Services (project nr: 33966).

### Measurements

**Five Facet Mindfulness Questionnaire** (FFMQ; Baer et al. 2006). The FFMQ uses a 5-point Likert response scale (1 = never or very rarely true, 5 = very often or always true). The higher the score on FFMQ the more mindful a person is and should thus experience less psychological problems. It contains 39 items, where each facet is represented with seven or eight items each. The five facets are: *observing*, *describing, acting with awareness*, *nonjudging of inner experience,* and *nonreacting to inner experience*. Baer et al. (2006) argue that using a total score of FFMQ to measure mindfulness will provide a distorted view of the relationships between mindfulness and other concepts, because the subscale scores of FFMQ measure different aspects of mindfulness that should not be collapsed into one. Thus, there is a need for clarifying the relation between the FFMQ facets. The Norwegian FFMQ has been validated for use in Norway (Dundas et al. 2013). In the current study, FFMQ showed adequate psychometric characteristics with Cronbach’s alpha on the five facets of 0.80, 0.88, 0.86, 0.92 and 0.80, respectively.

**Metacognitions Questionnaire 30** (MCQ-30; Wells and Cartwright-Hatton 2004). All subscales of the MCQ-30 have been shown to have positive relationships with obsessive-compulsive symptoms, pathological worry, and trait-anxiety. The MCQ-30 uses a 4-point Likert response scale (1 = do not agree, to 4 agree very much). Higher scores on MCQ represent higher levels of dysfunctional metacognitions. The five subscales are: *positive beliefs about worry*, *uncontrollability and danger*, *need to control thoughts*, *cognitive confidence*, and *cognitive self—consciousness*. MCQ *uncontrollability and danger* and MCQ *need to control thoughts* demonstrate particularly strong relationships with mood and anxiety disorders (Spada et al. 2008; Wells and Cartwright-Hatton, 2004). In the current study, MCQ-30 showed adequate psychometric characteristics with Cronbach’s alpha on the five subscales of 0.80, 0.85, 0.80, 0.88 and 0.85, respectively. The MCQ has been used with subscale factors as well as a total score as an indication of levels of metacognitions (e.g. Hjemdal et al. 2013).

**Patient Health Questionnaire 9-item** (PHQ-9; Spitzer et al. 1999). The PHQ-9 contains criteria for depression. The name PHQ-9 refers to the nine items in the questionnaire, and is based on the nine criteria for diagnosing depression in DSM-IV. Each item is reported on a four-point Likert scale (0 = not at all, 3 = almost every day), and the answers refer to the past two weeks. The PHQ-9 total score is used as a severity measure, and can range from 0 to 27. It has been shown to have good internal reliability and test-retest reliability, as well as criterion validity, construct validity and external validity (Kroenke et al. 2001). In the current study, the PHQ-9 showed adequate psychometric characteristics with a Cronbach’s alpha of 0.88.

**Generalized Anxiety Disorder 7-item** (GAD-7; Spitzer et al. 2006). GAD-7 is based on the DSM-criteria for generalized anxiety disorder. Each item is reported on a four-point Likert scale (0 = not at all, 3 = almost every day), and the answers refer to the past two weeks. The total score ranges between 0 – 21. It has been shown to have good reliability, as well as good criterion, construct, factorial and procedural validity. In the current study, the questionnaire showed adequate psychometric characteristics with a Cronbach’s alpha of 0.89.

**Obsessive-Compulsive Inventory Revised** (OCI-R; Foa et al. 2002). The OCI-R is an 18-item self-report questionnaire. OCI-R was developed to examine the presence and severity of obsessive-compulsive disorder (OCD) symptoms. Each item is rated on a 5-point Likert scale (0 = not at all, 4 = extremely). The higher the score the higher the level of OCD symptoms. The total score of the OCI-R provides information about the OCD severity, but there are also sub-scores which addresses the severity of the different types of obsessions and compulsions. There are six subscales included in the OCI-R: Washing, checking, obsessions, neutralizing, ordering and hoarding. The score of OCI-R ranges between 0 – 72, and higher scores indicate higher levels of OCD symptoms. The OCI-R has been shown to be a valid and reliable diagnostic tool (Foa et al. 2002). A study with a Norwegian sample found results supporting the validity of OCI-R (Solem et al. 2010). In the current study, the measure showed adequate psychometric characteristics with a Cronbach’s alpha of 0.90 for the total score.

### Statistics

Pearson correlation analyses were used to investigate the relationship between metacognition and mindfulness. The relationship between metacognition and mindfulness was further explored using Maximum Likelihood factor analysis with a direct oblimin rotation in which subscale scores of the MCQ and the FFMQ were used*.* After investigating the relationship between metacognition and mindfulness, the next step was to determine the independent predictors of symptoms using hierarchical regression analysis. We conducted three regression analyses. One using symptoms of anxiety as the dependent variable, the second using symptoms of depression, and the third using symptoms of OCD as the dependent variable. On the first step we entered gender and age, on the following steps we entered the factors extracted from the Maximum Likelihood analysis.

## Results

### Levels of depression, anxiety, and obsessive-compulsive symptoms in the sample

The mean score of PHQ-9 was 6.56 (SD = 5.61). The optimal cut-off score when using PHQ-9 is often set to 10 points (Manea et al. 2012). Using a cut-off point of 10 and above to describe the current sample; 20.5 % scored above this cut-off. With regard to severity; 11.1 % reported that their problems with depression made doing their work, taking care of things at home, or getting along with other people as “very difficult”, while 2.8 % reported this as being “extremely difficult”.

In this sample, the mean score of GAD-7 was 5.32 (SD = 4.61). The cut-off point for ‘caseness’ is a score of 10. In this sample, 14.8 % scored above cut-off. With regard to severity, 10.3 % reported that their anxiety problems made doing their work, taking care of things at home, or getting along with other people as being “very difficult”, while 2.3 % reported this as being “extremely difficult”.

The mean OCI-R score was 10.06 (SD = 9.78). The recommended cut-off point is 21, with scores at or above this level indicating the likely presence of OCD (Foa et al. 2002). In this sample, 11.6 % scored above cut-off. A summary of the scores on the different instruments are presented in Table 1.

**Table 1:** Levels of mindfulness, metacognitions, and symptoms (n = 224)

	Range	Mean	SD
MCQ			
*Positive beliefs about worry*	6 - 24	9.16	3.28
*Uncontrollability and danger*	6 - 24	11.04	4.66
*Cognitive confidence*	6 - 24	10.82	4.21
*Need to control thoughts*	6 - 24	9.50	3.88
*Cognitive self-consciousness*	6 - 24	12.59	3.87
Total	30 - 120	53.11	14.83
FFMQ			
*Observing*	8 - 40	24.25	6.07
*Describing*	8 - 40	28.89	6.50
*Acting with awareness*	8 - 40	27.27	5.91
*Nonjudging of inner exp.*	8 - 40	29.33	7.57
*Nonreacting to inner exp.*	7 - 35	19.93	5.12
PHQ-9	0 - 27	6.56	5.61
GAD-7	0 - 21	5.32	4.61
OCI-R	0 - 72	10.06	9.78

*Note. MCQ* Metacognitions Questionnaire, *FFMQ* Five Facet Mindfulness Questionnaire, *PHQ-9* Patient Health Questionnaire 9-item, *GAD-7* Generalized Anxiety Disorder 7-item, *OCI-R* Obsessive Compulsive Inventory-Revised

### Relationships between metacognition, mindfulness, and symptoms

The subscales *acting with awareness* and *nonjudging of inner experience* were the two facets of FFMQ with the strongest correlation with MCQ. Of these the *nonjudging of inner experience* dimension correlated most strongly with two MCQ subscales: *negative beliefs about uncontrollability* and *need to control thoughts*. These relationships, as with most of the associations between these two questionnaires were negative. They appear to show that the *nonjudging of inner experience* decreases as metacognitive *beliefs about the uncontrollability and danger of thoughts* and beliefs about the *need to control thoughts* increase. All of the five subscales in MCQ correlated positively and significantly (ranging from .23 to .71) with the symptom measures. The two subscales correlating the most with the three symptom measures were MCQ *uncontrollability and danger* and MCQ *need to control thoughts.* Four of the subscales in FFMQ correlated negatively and significantly (ranging from -.22 to -.65) with symptoms of emotional disorders while *observing* showed non-significant relationship with symptoms. The two mindfulness facets with the strongest correlations with symptoms were *acting with awareness* and *nonjudging of inner experience*.

The relationships between metacognition and mindfulness were explored using a Maximum Likelihood factor analysis in which subscale scores of the MCQ and the FFMQ were used. The aim of the factor analysis was to reveallatent constructs amongst the two sets of subscales. Two factors had eigenvalues above 1 and were extracted and subject to oblimin rotation. *Acting with awareness* and *nonjudging of inner experience* loaded onto a factor along with and dominated by the MCQ subscales and therefore appeared to reflect a *metacognition* factor*.* MCQ *cognitive self-consciousness* measures a construct primarily related to the first factor but it had a side-loading on the second factor. Because the variance is partially related to both factors it is difficult to interpret in further analyses in this particular context. The second factor consisted of three FFMQ subscales (*observing*, *nonreacting to inner experience*, and *describing*) and was labeled *mindfulness*. Standardized z-scores were used for the analysis and the FFMQ scales that corresponded with the metacognition factor were reversed when creating total scores for the two factors.

Partial correlations were used to examine unique relations between these latent factors and each symptom measure while controlling for symptom overlap. The metacognition factor showed strong zero-order correlations with all of the three symptom measures (ranging from .65 to .72) and correlations were still significant after controlling for other symptoms (ranging from .27 to .35). For the mindfulness factor, correlations with symptoms ranged from -.19 to -.27. Only the relationship with depression emerged as significant when controlling for symptom overlap in the partial correlation analyses. A summary of the correlation analyses and Maximum Likelihood factor analysis is presented in Table 2 and Table 3.

**Table 2:** Correlations between metacognitive subscales, mindfulness subscales, and symptom measures (n = 224)

				MCQ-30						
	GAD-7	PHQ-9	OCI-R	Positive beliefs	Negative beliefs	Cognitive confidence	Need to control thoughts	Cognitive self-consciousness	Total MCQ-30	*Age*
GAD-7				.31**	.71**	.38**	.58**	.47**	.68**	-.17**
PHQ-9	.73**			.23**	.62**	.45**	.59**	.34**	.62**	-.15*
OCI-R	.61**	.60**		.28**	.62**	.43**	.63**	.36**	.64**	*-.11*
FFMQ										
Observing	.10	.04	.04	.15*	.07	.07	.08	.38**	.20**	*.07*
Describing	-.22**	-.31**	-.28**	-.08	-.31**	-.32**	-.31**	.04	-.28**	.17**
Awareness	-.52**	-.60**	-.46**	-.23**	-.47**	-.44**	-.45**	-.30**	-.52**	.16*
Nonjudging	-.65**	-.55**	-.46**	-.37**	-.67**	-.30**	-.63**	-.52**	-.69**	.14*
Nonreact	-.36**	-.34**	-.24**	.05	-.35**	-.12	-.22**	.09	-.17*	*.04*
*Age*				-.21**	*-.11*	*-.11*	-.18**	-.15*	-.20**	

*Note. MCQ* Metacognitions Questionnaire, *FFMQ* Five Facet Mindfulness Questionnaire, *PHQ-9* Patient Health Questionnaire 9-item, *GAD-7* Generalized Anxiety Disorder 7-item, *OCI-R* Obsessive Compulsive Inventory-Revised. *p < 0.05, **p < 0.01

**Table 3:** Subscale loadings in the factor analysis with Maximum Likelihood and correlations between factors and psychiatric symptoms

	Factor 1 Metacognition		Factor 2 Mindfulness	
MCQ *Uncontrollability and danger*	.823		-.141	
FFMQ *Nonjudging of inner exp.*	-.805		-.050	
MCQ *Need to control thoughts*	.789		-.055	
FFMQ *Acting with awareness*	-.604		.034	
MCQ *Positive beliefs*	.510		.175	
MCQ *Cognitive confidence*	.480		-.090	
FFMQ *Observing*	.257		.652	
FFMQ *Nonreacting to inner exp.*	-.161		.625	
FFMQ *Describing*	-.263		.485	
	Correlations			
	Zero-order	Partial	Zero-order	Partial
GAD-7	.72**	.35**	-.21**	.03 ns
PHQ-9	.69**	.27**	-.27**	-.18**
OCI-R	.65**	.32**	-.22**	-.08 ns
Metacognition			-.19**	

*Note*. MCQ *Cognitive self-consciousness* is not included in the metacognition factor due to side-loading. Partial correlations control for the other symptom measures

### Predictors of psychological symptoms

Two separate factors emerged from the MCQ-30 and FFMQ in which metacognition could be separated from mindfulness and metacognition was a stronger predictor of specific psychological symptoms than mindfulness. The next step was to determine the independent predictors of symptoms using hierarchical regression analysis. We had two aims in doing this, first to provide some validation of the separate factors and more specifically test the incremental validity of mindfulness when controlling for metacognition. Such an analysis also permitted exploration of the individual predictors of a range of psychological symptoms.

### Predictors of anxiety

With GAD-7 as the dependent variable, the regression analysis revealed that at step one, age and gender explained 3 % of the variance where age was the significant predictor. The metacognition factor was entered on step 2 and explained 49 % of the variance in GAD-7 scores. The mindfulness factor was a non-significant predictor on step three. In the final step of the equation, metacognition was the only significant individual predictor of anxiety symptoms. A summary of this regression is presented in Table 4.

**Table 4:** Hierarchical regression analysis with GAD-7 as dependent variable (n = 224)

	*F* cha	*R* ^*2*^ cha	*β*	*t*
*Step 1*	3.61*	.03		
Gender			.05	.67
Age			-.17	−2.62**
*Step 2* Metacognition	221.60***	.49	.71	14.89***
*Step 3* Mindfulness	1.57	.00		
Gender			.03	.65
Age			-.03	-.63
Metacognition			.70	14.35***
Mindfulness			-.06	−1.25

*Note. GAD-7* Generalized Anxiety Disorder 7-item. * p < 0.05, ** p < 0.01, *** p < 0.001

### Predictors of depressive symptoms

The regression analysis with PHQ-9 as the dependent variable showed that at step one, age and gender explained 2 % with age being the significant predictor. When entering metacognition on step two an additional 46 % was explained and mindfulness on step 3 added a further 2 %. In the final step of the equation both metacognition and mindfulness were significant individual predictors of depressive symptoms. A summary of this regression is presented in Table 5.

**Table 5:** Hierarchical regression analysis with PHQ-9 as dependent variable (n = 224)

	*F* cha	*R* ^*2*^ cha	*β*	*t*
*Step 1*	2.69	.02		
Gender			.04	.55
Age			-.15	−2.26*
*Step 2* Metacognition	191.91***	.46	.69	13.85***
*Step 3* Mindfulness	9.95**	.02		
*Gender*			.04	.75
*Age*			.01	.13
*Metacognition*			.66	13.35***
*Mindfulness*			-.16	−3.15**

*Note. PHQ-9* Patient Health Questionnaire 9-item. * p < 0.05, ** p < 0.01, *** p < .001

### Predictors of obsessive-compulsive symptoms

Age and gender explained 1 % of OCD symptoms. Metacognition on step two added 42 %. Mindfulness on step three was a non-significant predictor. In the final step of the equation, metacognition was the only significant individual predictor of OCD symptoms. A summary of the regression is presented in Table 6.

**Table 6:** A multiple hierarchical regression analysis with OCI-R as dependent variable (n = 224)

	*F* cha	*R* ^*2*^ cha	*β*	*t*
*Step 1*	1.45	.01		
Gender			-.03	-.41
Age			-.11	−1.65
*Step 2* Metacognition	162.11***	.42	.66	12.73***
*Step 3* Mindfulness	2.97	.01		
Gender			-.04	-.75
Age			.04	.67
Metacognition			.65	12.28***
Mindfulness			-.09	−1.72

*Note. OCI-R* Obsessive Compulsive Inventory-Revised.*** p < .001

## Discussion

This study set out to explore the relationship between metacognitions and mindfulness and to examine how these constructs might relate to symptoms of depression, anxiety, and obsessive-compulsive disorder. Results of correlation analyses showed that metacognition was related to mindfulness as predicted by metacognitive theory. However, Maximum Likelihood factor analysis distinguished two clusters of traits. A metacognitive factor comprised of five MCQ subscales and two FFMQ subscales, and a narrower mindfulness factor comprised of three FFMQ subscales with a low side-loading from MCQ *cognitive self-consciousness*. The two factors were independently related to depression symptoms, but only the metacognition factor was related to anxiety and OCD symptoms. Metacognition was the strongest predictor of each set of psychological symptoms with little additional variance explained by the mindfulness factor. The negative association between mindfulness and depression scores in the regression suggests that higher depressive symptom levels are associated with lower levels of reacting, observing and describing events. However, these mindfulness components might reflect in part the symptoms of depression such as inactivity or disengagement from the environment. Further work is required to determine the distinctness of the mindfulness factor from depressive symptoms.

The Maximum Likelihood factor analysis revealed that metacognitive traits can be distinguished from mindfulness with three out of five mindfulness subscales (*observing*, *nonreacting to inner experience* and *describing*) showing weak relationships with metacognition. The metacognition factor had substantial loadings for all MCQ subscales with negative beliefs predominating. The negative loading of *nonjudging of inner experience* on the metacognition factor suggests that the tendency to evaluate one’s thoughts and feelings are more closely associated with metacognitive beliefs than with mindfulness. This pattern of findings supports a central assertion of the metacognitive model, that a syndrome of entanglement with thinking and analyzing events is linked to specific forms of metacognitive knowledge. It would seem that other dimensions of mindfulness (*observing, nonreacting to inner experience, and describing*) which are features of meditation practice may be only weakly related to the ideational processes that are considered most problematic in the metacognitive model. *Nonreacting to inner experience* was part of the mindfulness factor. Some *nonreacting to inner experience* items describe emotions and situations and how the person responds to them, whereas MCQ items refer to metacognitive knowledge, meaning knowledge about cognitions (not emotions/situations) and not about the person’s behavioral responses to them. These differences could explain why *nonreacting to inner experience* is not a part of the metacognition factor.

*Acting with awareness* also loaded negatively on the metacognition factor. The *acting with awareness* subscale appears to assess the ability to attend to what one is doing with items suggesting a good resistance to distraction. This loading is also parsimonious with the metacognitive model of emotional vulnerability, where the pattern of extended thinking (the cognitive attentional syndrome) that contributes to disorder is associated with reduced attentional flexibility (Wells and Matthews 1994, 1996). Moreover, the result is consistent with the use in metacognitive therapy of techniques such as *attention training* (Wells 1990) which is designed to increase flexibility and awareness of control.

Whilst the metacognitive dimension of *cognitive self-consciousness* loaded most highly on metacognition it also loaded on mindfulness. Examination of the bivariate subscale correlations reveals that this association is the result of shared variance with *observing* as there was no correlation with the other constituents of the mindfulness factor (*nonreacting to inner experience, describing*). *Observing* assesses the tendency to focus on thoughts, feelings and behaviors and therefore overlaps in content with *cognitive self-consciousness* which is the tendency to focus on thinking processes.

The mindfulness factor was composed of FFMQ subscales (*observing, nonreacting to inner experience, describing*) involving the concept of focusing on present-moment experience in an accepting way. This factor corresponds well to the definition of mindfulness presented in Buddhist-related applications (e.g. Kabat-Zinn 2003), but these features do not appear to be closely associated with psychological vulnerability. In contrast, metacognition was positively associated with each of the dimensions of psychological disorder symptoms measured and remained an independent predictor of all symptoms on the final step of the analysis. In contrast, mindfulness explained a small amount of additional variance in depression symptoms but not anxiety or OCD symptoms.

In summary, the present analysis indicated that the extent to which mindfulness is linked to maladaptation largely reflects metacognition. Specifically, higher negative beliefs about the *uncontrollability and danger of thoughts* and beliefs about *the need for control* loaded with high levels of *nonjudging of inner experience* and high levels of *acting with awareness*. Of the two emergent factors metacognition showed the strongest association with symptoms of psychological disorder. This factor corresponds to the Wells and Matthews (1994) metacognitive model of psychological disorder in which metacognitive beliefs coupled with extended conceptual processing and threat monitoring (the CAS) are central to emotional disorder. The results support the call to examine the structure and nature of mindfulness within the context of metacognitive theory (Wells, 2005) with a view to advancing the construct within a well specified psychological model.

### Strengths and limitations

The present study is innovative in examining how metacognitive traits relate to mindfulness constructs. The study used validated measures possessing good psychometric properties. A systematic review of instruments to measure self-reported mindfulness found that the FFMQ has the best psychometric properties (Park et al. 2013). However, there have also been criticisms of the scale (e.g. Van Dam et al. 2012) such as problems with combining positively and negatively worded items. Future studies should thus continue developing and improving the assessment of mindfulness.

Whilst the sample size was reasonable for this type of analysis, the cross-sectional nature of the data means that inferences about causality cannot be made. The data used was based on self-report, which means that it may have been biased by selective memory, personal experience and social desirability. For example, meditators and non-meditators complete the FFMQ differently (van Dam et al. 2009). It may be relevant to explore this in future studies. Also, the study did not measure detached mindfulness as described in Wells’ metacognitive theory. As such the study cannot address the relationship between mindfulness and detached mindfulness.

Measures of anxiety and depression were chosen as symptom measures because they are the most frequent emotional disorders in the population. A measure of OCD was also included as mindfulness and metacognitions have been implicated as important in OCD, however, other measures of other emotional disorders could have been included as well and a measure of global functioning would be a reasonable addition to the study. To ease burden on participants we chose to include a few brief symptom measures. Future studies should look into the relationship between mindfulness and metacognition on other symptom measures as well as measures of global functioning.

A major limitation is that convenience sampling was used and limited information about the sample is available, for example we do not know if any of the participants were treatment seeking. The uncertainty with regard to the representativeness of the sample means that we do not know if the latent structure of relationships between subscales applies in a clinical group. Several variables showed significant skewness and kurtosis and generally this would indicate that the results of the analyses should be interpreted with some caution. A single study does not implicate changes to theories of treatment. However, if further studies replicate the findings from our study using patient populations and more rigorous designs, there would be indications as to which components of mindfulness and metacognitions are most important to address in therapy.

## Conclusions

In conclusion, the results of the present study suggest that mindfulness and metacognition traits as assessed by the FFMQ and MCQ-30 can be described by two latent factors. A large metacognition factor that correspond with the Wells and Matthews (1994) metacognitive model of dysfunctional processing and a smaller mindfulness factor that corresponds more with Buddhist-based conceptions of an experiential stance of observational acceptance. Psychological disorder symptoms were most strongly associated with the metacognition factor with little or no contribution made by mindfulness. These results imply that mindfulness as assessed by constructs such as the FFMQ is rather heterogeneous in its constituent parts, a result that is consistent with definitions of the construct that appear to combine attention and cognitive control (metacognition) with a direct experience and acceptance of the present. However, some of the most characteristic and unique feature of mindfulness; *focusing on present moment experience* may well be the least important from the perspective of psychological wellbeing. The results underscore the need for a greater psychological theory informed analysis.

## Abbreviations

CAS: Cognitive attentional syndrome
FFMQ: Five facet mindfulness questionnaire
GAD-7: Generalized anxiety disorder 7-item
MCQ-30: Metacognitions questionnaire 30
OCD: Obsessive-compulsive disorder
OCI-R: Obsessive-compulsive inventory revised
PHQ-9: Patient health questionnaire 9-item
